# Vascular Endothelial Growth Factor Receptor 2-Targeted Therapy Suppresses the Progression of Alpha-Fetoprotein-Positive Hepatocellular Carcinoma After Combination Therapy With Anti–Programmed Death-Ligand 1 and Anti–Vascular Endothelial Growth Factor-A Antibodies

**DOI:** 10.1016/j.gastha.2025.100778

**Published:** 2025-09-01

**Authors:** Gen Sugiyama, Kouki Nio, Hikari Okada, Akihiko Kida, Keisuke Sako, Yasunori Iwata, Hideo Takayama, Yutaro Kawakami, Tomoyoshi Chiba, Kazuki Nagai, Saiho Sugimoto, Masaki Nishitani, Tomoyuki Hayashi, Hajime Takatori, Tetsuro Shimakami, Masao Honda, Taro Yamashita

**Affiliations:** 1 Department of Gastroenterology, Graduate School of Medicine, Kanazawa University, Kanazawa, Japan; 2 Department of Nephrology and Rheumatology, Graduate School of Medicine, Kanazawa University, Kanazawa, Japan

**Keywords:** Cancer Immunotherapy, Cancer Stem Cell, Tumor Microenvironment

## Abstract

**Background and Aims:**

Combination therapy with the anti–programmed death-ligand 1 (anti-PD-L1) antibody atezolizumab and the anti–vascular endothelial growth factor-A (anti-VEGF-A) antibody bevacizumab (Atezolizumab/Bevacizumab) has commonly been used as first-line treatment for advanced hepatocellular carcinoma (HCC). However, effective second-line treatment options remain under debate. The anti–vascular endothelial growth factor receptor 2 (VEGFR2) antibody ramucirumab has shown promise in unresectable HCC with high serum alpha-fetoprotein (AFP) levels, but its efficacy after Atezolizumab/Bevacizumab is unclear. This study investigated the effects of VEGFR2 inhibition on the tumor microenvironment and cancer stem cells (CSCs) in HCC after anti-PD-L1/anti-VEGF-A treatment.

**Methods:**

AFP-positive human and mouse HCC cell lines were used to evaluate the effects of the antimouse VEGFR2 antibody DC101. Syngeneic mouse models were employed to analyze the impact of DC101 as a secondary treatment following anti-PD-L1/anti-VEGF-A treatment. Various molecular biological analyses were conducted to assess tumor growth, gene expression, cellular localization, cell–cell interactions, and alterations in the tumor microenvironment.

**Results:**

DC101 significantly inhibited tumor growth and disrupted cell–cell interactions between AFP-positive HCC cells and VEGFR2-positive endothelial cells (ECs) in human HCC xenograft models. DC101 treatment following anti-PD-L1/anti-VEGF-A treatment showed notable antitumor effects in a syngeneic mouse model, with reduced expression of CSC and EC markers. Comprehensive gene expression analysis revealed that DC101 downregulates pathways associated with cancer stemness. Furthermore, single-cell analysis demonstrated that DC101 suppresses CSCs by disrupting their interaction with ECs and induces alterations in the tumor immune microenvironment.

**Conclusion:**

VEGFR2-targeted therapy not only suppressed tumor angiogenesis but also inhibited CSCs and enhanced antitumor immune activity, suggesting its potential utility as a second-line treatment following Atezolizumab/Bevacizumab.

## Introduction

Liver cancer is considered the third leading cause of cancer death worldwide, with hepatocellular carcinoma (HCC) accounting for 75%–85% of cases.^1^ In recent years, combination therapy primarily comprising cancer immunotherapy and multi–tyrosine kinase inhibitors (TKIs) targeting the vascular endothelial growth factor (VEGF) signaling pathway has been established as the standard treatment for HCC. Among these, atezolizumab, an antiprogrammed death-ligand 1 (anti-PD-L1) antibody, plus bevacizumab, an anti-VEGF-A antibody, combination therapy (Atezolizumab/Bevacizumab) has been widely used as the first-line treatment choice.^2^ However, there is still no established consensus on second-line treatment options after Atezolizumab/Bevacizumab.

Ramucirumab, an anti–vascular endothelial growth factor receptor 2 (VEGFR2) antibody, has demonstrated efficacy in unresectable HCC with serum alpha-fetoprotein (AFP) levels of ≥400 ng/mL that is refractory or intolerant to sorafenib.^3^ Ramucirumab is the only molecular targeted therapy in the pharmacotherapy of HCC to have shown utility in patient selection based on a biomarker. In recent, Shimose et al. reported in a retrospective study that the therapeutic effect of ramucirumab as a post-Atezolizumab/Bevacizumab treatment was significantly greater in terms of progression-free survival than when ramucirumab was administered following other therapies,^4^ the molecular mechanisms of its efficacy as a second-line treatment following Atezolizumab/Bevacizumab is not elucidated.

Previously, we proposed a molecular classification of HCC based on the gene and protein expression status of serum AFP and the hepatic stem cell marker epithelial cell adhesion molecule (EpCAM).^5^^,^^6^ Among these subtypes, hepatic stem cell-like HCC, characterized by positivity for AFP and EpCAM, was identified as a subtype with a strong tendency for vascular invasion and poor prognosis. Furthermore, we found that EpCAM-positive HCC cells exhibit features of cancer stem cells (CSCs), which are believed to play essential roles in cancer initiation, maintenance, drug resistance, and distant metastasis, as they express high levels of AFP. CSCs are abundant in the invasive front of tumors, where stromal cells rich in endothelial cells (ECs), fibroblasts, and immune cells are present, forming a tumor microenvironment (TME) that maintains the properties and survival of CSCs.^7^^,^^8^ Within the TME, a vascular niche has been reported to not only supply blood to tumor cells but also support cancer stemness via VEGF.^9^^,^^10^ These findings suggest the possibility that ramucirumab inhibits the vascular niche of AFP-positive liver CSCs and may not only inhibit angiogenesis but might also impede the maintenance of cancer stemness. In this study, we investigated the antitumor effects of anti-VEGFR2 antibody inhibition in HCC after anti-PD-L1/anti-VEGF-A treatment and examined the alteration of the TME constructed by CSCs.

## Methods

### Cell Lines and Reagents

The human HCC cell lines Huh1, Huh7, HLF, and SK-Hep-1, and human umbilical vein endothelial cell (HUVEC) were obtained from the Japanese Collection of Research Bioresources Cell Bank (Osaka, Japan) or American Type Culture Collection (Manassas, VA), while the HCC cell line MT was established from resected HCC specimens as described previously.^11^ The subject gave his/her written informed consent for inclusion before his/her participated in the study in which the tissue would be used for future study (2016-093, October 25, 2011). The study was conducted in accordance with the Declaration of Helsinki, and the protocols were approved by the Ethics Committee of the Graduate School of Medical Sciences, Kanazawa University.

The mouse HCC cell line PP53-2 was established by breeding Alb-promoter human PDGF-C transgenic mice with Alb-cre/Trp53flox/flox mice to generate liver-specific human PDGF-C transgenic and Trp53 knockout mice. Liver tumor tissues were excised from 52-week-old liver-specific human PDGF-C transgenic and Trp53 knockout mice, and cell suspensions were prepared using a gentleMACS Dissociator (Miltenyi Biotec, Bergisch Gladbach, Germany) and Mouse Tumor Dissociation Kit (130-096-730; Miltenyi Biotec). The cell suspension was cultured in a 10-cm dish, and the cells were passaged 5–10 times to remove nonparenchymal cells. Subsequently, they were subcutaneously transplanted into nonobese diabetic/severe combined immunodeficiency (NOD/SCID) mice. After subcutaneous tumor formation, the cells were again made into a suspension, and the process of cell culture and subcutaneous transplantation was repeated twice. Among the cell lines for which cell culture was possible, AFP-positive/EpCAM-positive mouse HCC cells were named pp53-2 cells. The HCC cells were maintained in Dulbecco’s modified Eagle medium (DMEM) supplemented with 5% fetal bovine serum (Gibco, Grand Island, NY) at 37 °C. HUVECs were maintained in Endothelial Cell Growth Medium-2 BulletKit (Lonza, Basel, Switzerland).

The antihuman VEGFR2 antibody ramucirumab (CYRAMZA) was purchased from Eli Lilly and Company (Indianapolis, IN). The antimouse PD-L1 antibody (clone B7-H1) was purchased from Bio X Cell (Lebanon, NH) and the antimouse VEGF-A antibody (clone 2G11-2A05) was purchased from BioLegend (San Diego, CA). The antimouse VEGFR2 antibody DC101 was provided by Eli Lilly and Company.

### Huh1/HUVEC Coculture Assay

A total of 2×10^5^ Huh1 cells were cultured in DMEM in 6-well plates and 1×10^5^ HUVECs were plated in 6-well Falcon culture insert (Corning, Corning, NY) in Endothelial Cell Growth Medium-2 BulletKit treated with 1 μg/mL or 10 μg/mL ramcirumab. After 72 hours of coculture, RNA was extracted from Huh1 cells.

### Immunohistochemistry and Immunofluorescence

Antimouse VEGFR2 monoclonal (#2479; Cell Signaling Technology, Danvers, MA), antihuman AFP monoclonal (#422221; Nichirei Biosciences, Inc, Tokyo, Japan), antimouse EpCAM monoclonal (ab213500; Abcam, Cambridge, UK), and antimouse CD34 monoclonal (ab8158; Abcam) antibodies were used as primary antibodies for immunohistochemistry or immunofluorescence staining. For immunohistochemistry, an EnVision + Kit (Dako, Carpinteria, CA) was used to detect primary antibodies qualitatively. For immunofluorescence, Alexa Fluor 488-conjugated antirat IgG and Alexa Fluor 594-conjugated antirabbit IgG (Invitrogen, Carlsbad, CA) were used as secondary antibodies. All images were obtained using an inverted microscope (BZ-X800; Keyence, Osaka, Japan). Relative comparisons of the immunohistochemical staining area and cell-to-cell distances were performed using ImageJ (version 1.8.0; National Institutes of Health, Bethesda, MA).

### Flow Cytometry Analysis

The cells were resuspended in Hank’s balanced salt solution (Lonza) supplemented with 1% HEPES and 2% fetal bovine serum and incubated with an allophycocyanin-conjugated antimouse CD326 (EpCAM) antibody (#118214; BioLegend) or FITC-conjugated antimouse CD90 antibody (FAB7335G; R&D Systems, Minneapolis, MN) on ice for 30 min. After labeling, the cells were assessed using a BD Accuri C6 Plus Flow Cytometer (BD Biosciences, San Jose, CA). Data analysis was performed using FlowJo Software (version 10.10.0; BD Biosciences).

### Real-Time Quantitative PCR

Total RNA was isolated from mouse tumor tissue using an RNeasy Mini Kit (Qiagen, Hilden, Germany). Quantitative PCR probes for *EpCAM* (Hs00158980_m1), *AFP* (Hs00173490_m1), *VEGFA* (Hs00900055_m1), *VEGFB* (Hs00173634_m1), *VEGFC* (Hs01099203_m1), *FLT1* (Hs01052935_m1), *KDR* (Hs00911700_m1), *Epcam* (Mm00493214_m1), *Afp* (Mm00431715_m1), *Cd34* (Mm00519283_m1), *Pecam1* (Mm00476702_m1), and *Pdgfb* (Mm01298578_m1) were procured from Applied Biosystems (Foster City, CA). Gene expression analysis was conducted in triplicate using the QuantStudio 12K Flex System (Applied Biosystems). Expression levels of the target genes were normalized to a reference gene (*ACTB* or *ActB*).

### Microarray Analysis

Total RNA was isolated from mouse tumor tissue using an RNeasy Mini Kit (Qiagen). Comprehensive gene expression analysis was performed using a Mouse Clariom S Array (Thermo Fisher Scientific, Waltham, MA). Normalization of the microarray data was performed using a summarization algorithm (Exon SST-RMA without stabilization), and quality control was subsequently assessed through hierarchical clustering and principal component analysis. Genes altered by > 1.4-fold compared to controls were visualized using GENESIS software (version 1.8.1). Signaling pathways significantly downregulated by DC101 (*P* < .01) compared to the control were identified. *P* values were transformed to their negative logarithms (-log) and visualized in GraphPad Prism (version 9.5.1, GraphPad Software, San Diego, CA) according to their significance levels. Student’s *t*-test was used to compare gene expression in the treatment groups using Graphpad Prism.

### Western Blotting

Tumor tissues were lysed in radioimmunoprecipitation assay buffer as described previously.^12^ The primary antibodies used for western blotting were an anti-EpCAM polyclonal antibody (ab213500; Abcam) and anti–β-actin antibody (sc-47778; Santa Cruz Biotechnology, Dallas, TX). Immune complexes were visualized using enhanced chemiluminescence detection reagents (Amersham Biosciences Corp., Piscataway, NJ).

### Single-Cell Multiplex Staining Analysis

Formalin-fixed, paraffin-embedded tissues were used for single-cell multiplex staining. To measure the TME, PhenoCycler (Akoya Biosciences, Marlborough, MA), a system that can use many antibodies at once and produces single-cell fractions, was utilized. Formalin-fixed, paraffin-embedded biopsies were sectioned on poly-L-lysine-coated square glass coverslips (22 × 22 mm). Fluorescent oligonucleotides (Akoya Biosciences) were conjugated to antibodies for EpCAM (ab213500; Abcam), VE-cadherin (555289; BD Biosciences), CD8 (ab230156,;Abcam), programmed cell death protein 1 (PD1) (ab228857; Abcam), and T cell immunoreceptor with Ig and ITIM domains (TIGIT) (ZRB1454; Sigma-Aldrich, St. Louis, MO) using CODEX Conjugation Kit (Akoya Biosciences). Mouse FFPE IO panel were purchased from Akoya Biosciences. Slides were photographed using the PhenoCycler system and imaged using the PhenoCycler Processor (version 1.8) platform (Akoya Biosciences), and the cells were segmented with DAPI. Visualization, classification of cell populations, quantification of classified cell numbers, and cell neighborhood analysis were performed using QuPath (Akoya Biosciences).

### Animal Studies

NOD.CB17-Prkdcscid/J (NOD/SCID) mice and C57BL/6J mice were purchased from The Jackson Laboratory Japan, Inc (Yokohama, Japan) and housed under specific pathogen-free conditions. Huh1, MT or SK-Hep-1 cells (1.0 × 10^6^ cells) were resuspended in 200 μL of a 1:1 DMEM:Matrigel (BD Biosciences) mixture and injected subcutaneously into 6-week-old NOD/SCID mice. Once the tumors had reached a measurable size, the mice were divided randomly into two groups and injected intraperitoneally with phosphate-buffered saline or 40 mg/kg DC101 twice a week for 10–15 days. PP53-2 cells (1.0 × 10^6^ cells) were resuspended in 200 μL of a 1:1 DMEM:Matrigel mixture and injected subcutaneously into 6-week-old C57BL/6J mice. The mice were injected intraperitoneally with 10 mg/kg antimouse PD-L1 and 5 mg/kg antimouse VEGF-A twice a week for 2 weeks or 3 weeks, starting at 1–2 week after cell transplantation. Once the tumors had reached a measurable size, the mice were divided randomly into two groups and injected intraperitoneally with phosphate-buffered saline or 40 mg/kg DC101 twice a week for 5 weeks. The size of subcutaneous tumors was recorded once or twice a week. The tumor and liver tissues were promptly collected after euthanizing the mice. Formalin-fixed, paraffin-embedded liver tissues were stained with hematoxylin and eosin (H&E) to evaluate the presence of liver metastases. The experimental protocol was approved by the Kanazawa University Animal Care and Use Committee and conformed to the Guide for the Care and Use of Laboratory Animals prepared by the National Academy of Sciences.

### Serum Creatinine Measurement and Renal Glomerular Assessment

Mouse serum creatinine levels were measured using the EnzyChrom Creatinine Assay Kit (BioAssay Systems, Hayward, CA) according to the manufacturer’s instructions. Formalin-fixed, paraffin-embedded kidney tissues were stained with H&E. After blinding the samples, glomerular assessment was performed by two nephrologists.

### Statistical Analysis

An unpaired *t*-test or chi-square test was performed using Prism (version 9.5.1; GraphPad Software). Statistical significance was defined as a *P* value < .05.

## Results

### DC101 Suppresses Tumor Growth with the Inhibition of Cell–Cell Interactions Between AFP-Positive Human HCC Cells and VEGFR2-Positive Mouse Vascular ECs in Human HCC Xenograft Models

**Fig. 1:**
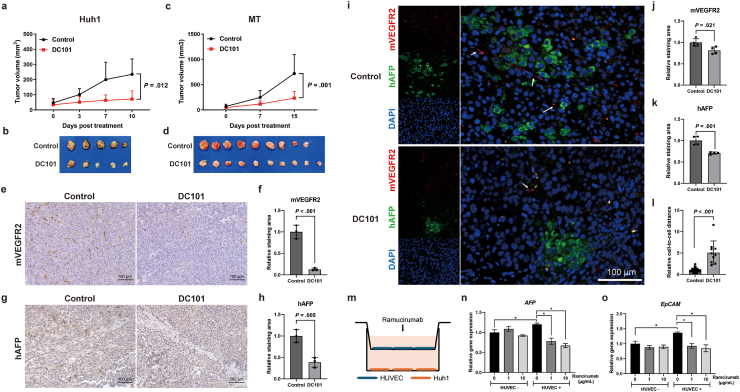
(A-D) Antitumor effect of DC101 for Huh1 tumor (A) (B) and MT tumor (C) (D). (E) Mouse VEGFR2 expression in tumor tissue of control and DC101 treatment. (F) Relative staining area of mouse VEGFR2 in tumor tissues. (G) Human AFP expression in tumor tissue of control and DC101 treatment. (H) Relative staining area of human AFP in tumor tissues. (I) Representative photomicrographs showing the proximity of AFP-positive human HCC cells and VEGFR2-positive mouse ECs in MT xenograft tumors after treatment. White arrows show mouse ECs. Red: mouse VEGFR2, green: human AFP, blue: DAPI. (J) Relative staining area of mouse VEGFR2 in tumor tissues. (K) Relative staining area of human AFP in tumor tissues. (L) Relative cell-to-cell distances from human AFP-positive cells to mouse VEGFR2-positive cells. (M) Schematic diagram of the Huh1 and HUVEC coculture experiment. (N-O) Relative gene expression in Huh1 cells 72 hours after coculture with HUVECs and Ramucirumab treatment; *AFP* (N), *EpCAM* (O).

We first confirmed the antitumor effect of the antimouse VEGFR2 antibody DC101 by inhibiting angiogenesis using a xenograft mouse model transplanted with AFP-positive/EpCAM-positive human HCC cells. We found that DC101 significantly inhibited tumor growth in the Huh1 (Figure 1A and B) and MT (Figure 1C and D) xenograft models. Immunohistochemical analysis of the tumor tissues revealed that, as expected, mouse VEGFR2-positive ECs in the tumor tissues were significantly suppressed by DC101 (Figure 1E and F). Furthermore, intriguingly, human AFP-positive cells, which exhibit characteristics of stem cell-like HCC, were also significantly reduced (Figure 1G and H). In addition, immunofluorescence staining demonstrated that DC101 not only reduced both mouse VEGFR2-positive ECs and human AFP-positive HCC cells (Figure 1I–K) but also disrupted the proximity of human AFP-positive HCC cells to mouse VEGFR2-positive ECs (Figure 1L). We further investigated cell–cell interactions using a coculture system of HCC cells and HUVECs with a cell culture insert (Figure 1M). Coculture with HUVECs enhanced the expression of AFP and EpCAM in HCC cells, whereas DC101 suppressed the stemness-promoting effects induced by this interaction (Figure 1O and P).

**Fig. 2:**
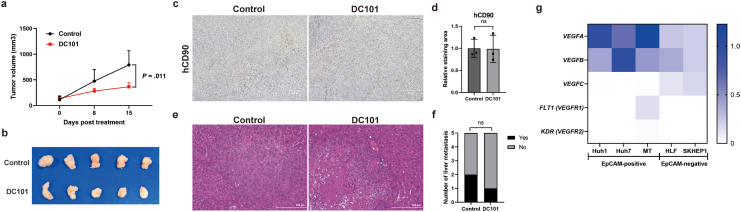
(A, B) Antitumor effect of DC101 for SK-Hep-1 tumor. (C) Human CD90 expression in tumor tissue of control and DC101 treatment. (D) Relative staining area of human CD90 in tumor tissues. (E) Representative liver metastasis of control and DC101 treated mouse. (F) Presence rate of liver metastasis in control and DC101 treated mice. (G) Relative VEGFs and VEGF receptors gene expressions in EpCAM-positive and EpCAM-negative HCC cell lines. The VEGFA gene expression in Huh1 was set to 1.

Next, we investigated the effect of DC101 on AFP-negative/EpCAM-negative human HCC cell line, SK-Hep-1 cells. We found that DC101 inhibited tumor growth in SK-Hep-1 cells, similar to AFP-positive HCC cells. However, the number of mesenchymal CD90-positive cells, a representative AFP-negative liver CSC population, did not decrease, nor was their characteristic metastatic potential suppressed (Figure 2A–F). To examine the differential effects of DC101 on each CSC subtype, we further analyzed VEGF gene expressions in both AFP-positive and AFP-negative HCC cells. Interestingly, the expression levels of *VEGFA* and *VEGFB* were higher in AFP-positive cells compared to AFP-negative cells. In contrast, the expression levels of *FLT1* (*VEGFR1*) and *KDR* (*VEGFR2*) were very low in both cell types (Figure 2G). These results support the hypothesis that AFP-positive HCC cells have stronger interactions with ECs, and that inhibiting VEGFR2-positive ECs disrupts this interaction.

Taken together, these findings suggest that DC101 not only simply inhibited angiogenesis but also may have the potential of exerting antitumor effects by reducing AFP-positive/EpCAM-positive HCC CSCs and inhibiting the cell–cell interactions between AFP-positive/EpCAM-positive HCC CSCs and ECs.

### Sequential DC101 Treatment following anti-PD-L1 and anti-VEGF-A Combination Therapy Shows a Significant Antitumor Effect in a Syngeneic Mouse Model

**Fig. 3:**
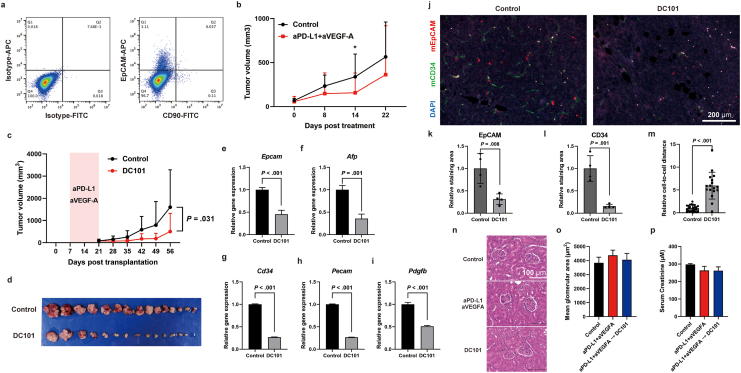
(A) Expression of the stem cell markers EpCAM and CD90 in the established mouse HCC cell line PP53-2. (B) Antitumor effect of control or anti-PD-L1 antibody (aPD-L1) and anti-VEGF-A antibody (aVEGF-A) combination therapy in a syngeneic mouse model. (C, D) Antitumor effect of sequential DC101 treatment after combination therapy in a syngeneic mouse model. (D-H) Relative gene expression in tumor tissue of control and DC101 treatment; *Epcam* (E), *Afp* (F), *Cd34* (G), *Pecam* (H), *Pdgfb* (I). (J) Representative photomicrographs showing EpCAM-positive CSCs and CD34-positive ECs in syngeneic mouse tumors after treatment. Red: mouse EpCAM, green: mouse CD34, blue: DAPI. (K) Relative staining area of mouse EpCAM in tumor tissues. (L) Relative staining area of mouse CD34 in tumor tissues. (M) Relative cell-to-cell distances from mouse EpCAM-positive cells to mouse CD34-positive cells. (N) Representative glomerular images from mice treated with control, combination therapy, or DC101 following combination therapy. (O) Mean glomerular area in mice treated with control, combination therapy, or DC101 following combination therapy. (P) Serum creatinine levels in mice treated with control, combination therapy, or DC101 following combination therapy. ∗*P* < .05.

Next, we verified the usefulness of the antimouse VEGFR2 antibody DC101 as a secondary treatment following anti-PD-L1 and anti-VEGF-A combination therapy, which is the current standard first-line treatment for HCC. To verify its usefulness, we first established an HCC syngeneic model using the established mouse HCC cell line PP53-2. By hepatic stem cell expression analysis using flow cytometry, PP53-2 was found to be an HCC cell line with EpCAM-positive CSCs and a small population of CD90-positive CSCs (Figure 3A). After 2 weeks of combination immunotherapy in an HCC syngeneic model transplanted with PP53-2 cells, we administered DC101 sequentially for 5 weeks. In this HCC syngeneic model, the loss of tumor suppression by the third week of combination immunotherapy suggests that switching treatments after 2 weeks is a reasonable approach to evaluate the therapeutic effect on resistant tumors (Figure 3B). We found that DC101 significantly suppressed tumor growth compared to the control (Figure 3C and D). Gene expression analysis of tumor tissue revealed that not only the expression of vascular endothelial markers such as *Cd34*, *Pecam*, and *Pdgfb* but also *Epcam* and *Afp* expression were decreased by DC101 treatment (Figure 3E–I). In addition, immunofluorescence staining demonstrated that DC101 disrupted cell–cell proximity, along with reducing the expression of EpCAM and CD34 (Figure 3J–M). In clinical settings, long-term VEGF inhibition is associated with glomerular injury; however, in this study, DC101 treatment following combination immunotherapy did not induce significant serum creatinine elevation and glomerular damage (Figure 3N–P).

### DC101 Suppresses the Cancer Stemness of EpCAM-Positive HCC

**Fig. 4:**
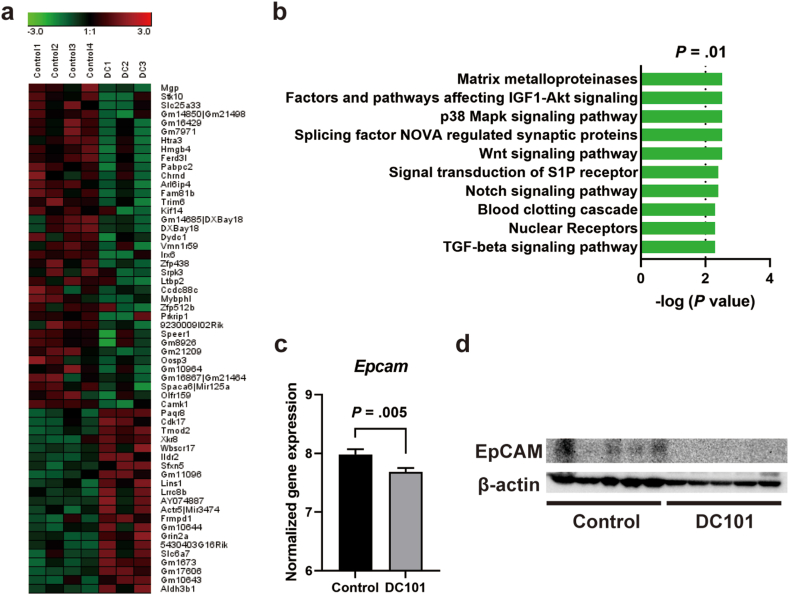
(A) Upregulated/downregulated genes with >1.4-fold difference in expression between control (*n* = 4) and DC101-treated (*n* = 3) mouse tumor tissues. Red and green cells depict high and low expression levels, respectively. (B) Top 10 signaling pathways downregulated significantly (*P* < .01) by DC101 treatment are shown. (C) Normalized *Epcam* gene expression in control and DC101-treated mouse tumor tissues. (D) Western blot analysis of EpCAM and β-actin in control and DC101-treated mouse tissues.

To examine the impact of DC101 on gene expression and signaling pathway alterations in EpCAM-positive HCC, we performed a comprehensive microarray-based analysis. Genes that showed increase or decrease in expression of >1.4-fold following DC101 treatment are shown in Figure 4A. No statistically significant differences were observed for these genes in the moderated t-test with multiple testing correction. Interestingly, the most highly downregulated gene among the 37 genes downregulated by DC101 treatment was *Mgp*, which has recently been reported to be associated with T-cell exhaustion.^13^ Pathway analysis using the genes altered by DC101 indicated that signaling pathways involved in the regulation of liver cancer stemness, such as Wnt, Notch, and TGF-beta,^14^ were significantly downregulated following DC101 treatment (Figure 4B). In gene expression analysis using a microarray, *Epcam* expression was also suppressed by DC101 treatment (Figure 4C). Western blotting was additionally performed to assess cancer stemness in tumor tissues after DC101 treatment, revealing decreased expression of EpCAM (Figure 4D). These data suggest that DC101 exerts its antitumor effects by suppressing the cancer stemness of EpCAM-positive HCC.

### DC101 Reduces the Number of EpCAM-Positive Cancer Stem Cells and VE-Cadherin-Positive Vascular ECs and Alters the Tumor Immune Microenvironment

**Fig. 5:**
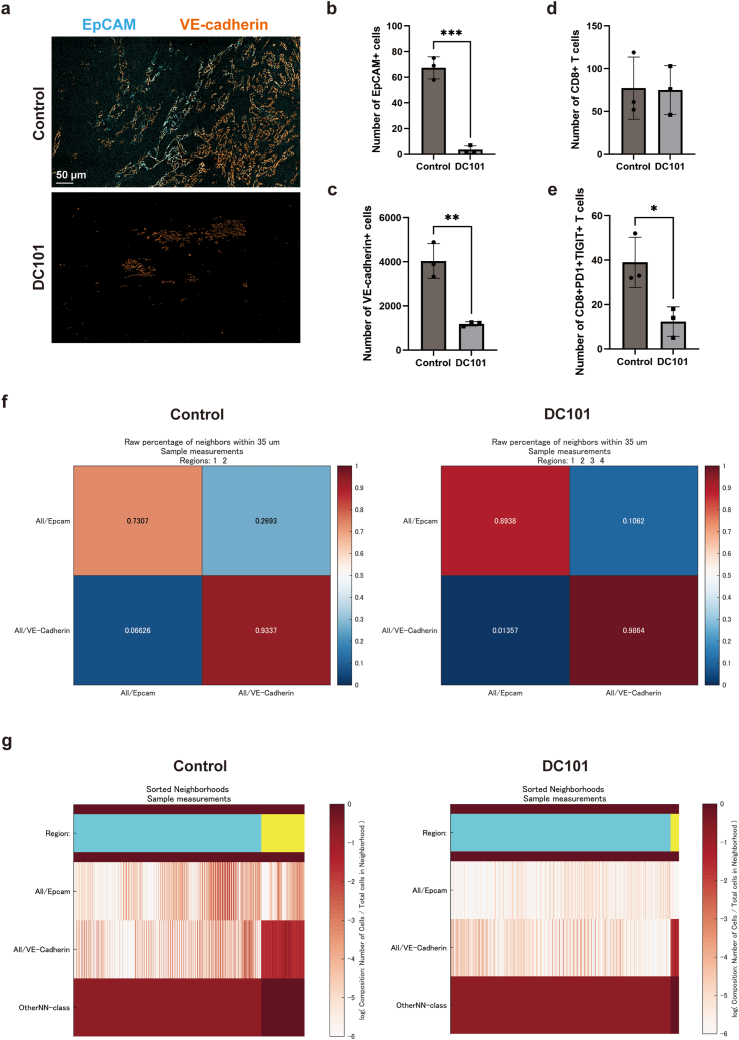
(A) Representative multiplexed microscopy images using posttreatment mouse tumor tissues. Cyan: EpCAM, orange: VE-cadherin. (B-C) Component cell number of posttreatment mouse tumor tissues; EpCAM-positive cells (B), VE-cadherin-positive cells (C), CD8-positive T cells (D), PD1-positive/TIGIT-positive CD8 T cells (E). ∗*P* < .05, ∗∗*P* < .01, ∗∗∗*P* < .01. (F) Raw percentage of neighbors within 35 μm in EpCAM-positive or VE-cadherin-positive cells in tumors after control or DC101 treatment. (G) Sorted neighborhoods in EpCAM-rich region or VE-Cadherin-rich region in tumors after control or DC101 treatment.

We further analyzed the impact of DC101 treatment on the TME by examining the cellular composition of the TME using single-cell multiplex staining analysis. As expected, DC101 significantly reduced the number of EpCAM-positive cells and VE-cadherin-positive cells (Figure 5A–C). Interestingly, while DC101 did not affect the infiltration of total CD8-positive T cells within the tumor (Figure 5D), it reduced the number of exhausted CD8 T cells that were PD1-positive and TIGIT-positive (Figure 5E).

We then examined the spatial proximity between EpCAM-positive cells and VE-cadherin-positive cell populations. DC101 treatment decreased the proportion of EpCAM-positive cells and VE-cadherin-positive cells located within 35 μm of each other (Figure 5F), and cell neighborhood analysis also revealed a disruption of the spatial proximity between EpCAM-positive CSCs and VE-cadherin-positive ECs. (Figure 5G).

**Fig. 6:**
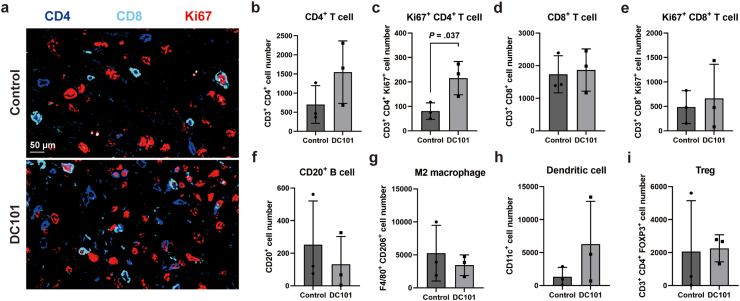
(A) Representative multiplexed microscopy images using posttreatment mouse tumor tissues. Blue: CD4, cyan: CD8, red: Ki67. (B-I) Component cell number of posttreatment mouse tumor tissues; CD4-positive T cells (B), Ki67-positive/CD4-positive T cells (C), CD8-positive T cells (D), Ki67-positive/CD8-positive T cells (E), CD20-positive B cells (F), F4/80-positive/CD206-negative M1 macrophages (G), F4/80-positive/CD206-positive M2 macrophages (H), FoxP3-positive/CD4-positive regulatory T cells (Treg) (I).

Subsequently, the tumor immune microenvironment was analyzed by single-cell multiplex staining using the Mouse FFPE IO panel. As a result, DC101 treatment significantly increased the intratumoral infiltration of Ki67-positive CD4^+^ T cells. In contrast, no significant changes were observed in the number of CD8^+^ T cells (including Ki67-positive cells), B cells, or immunosuppressive cells such as M2 macrophages, dendritic cells, and regulatory T cells (Tregs) (Figure 6A–I). These data suggest that DC101 may induce alterations in the immune microenvironment within the TME, in addition to reducing ECs and CSCs in EpCAM-positive HCC.

## Discussion

In our previous studies on the molecular classification of HCC using hepatic stem cells, AFP-positive HCC was strongly associated with EpCAM-positive epithelial stem type HCC.^5^^,^^6^ This subtype is characterized by high tumor invasiveness and poor prognosis, indicating a highly malignant form of HCC, which is similarly classified in the molecular biological classifications of Hoshida et al.^15^ and Boyault et al.^16^ Ramucirumab, an anti-VEGFR2 antibody, has demonstrated efficacy in patients with HCC who have high serum AFP levels^3^; however, the mechanism by which it inhibits tumor progression in highly malignant HCC has not been fully elucidated. Therefore, we first examined the effects of inhibiting VEGFR2 on intratumoral ECs and AFP-positive CSCs. Interestingly, when DC101, an antimouse VEGFR2 antibody, was administered to a human HCC patient-derived xenograft mouse model, we observed not only an inhibition of mouse ECs but also a reduction in human AFP-positive HCC cells. Moreover, it was revealed that the proximity between ECs and AFP-positive HCC cells was disrupted. The expression levels of *VEGFA* and *VEGFB* were higher in AFP-positive cells compared to AFP-negative cells. This support the hypothesis that AFP-positive HCC cells have stronger interactions with ECs, and that inhibiting VEGFR2-positive ECs disrupts this interaction.These findings also suggest a crucial role for ECs in the TME in supporting the survival of CSCs. Indeed, the importance of crosstalk between tumor blood vessels and CSCs in tumor progression via VEGF signaling has been reported.^13^ Therefore, based on the results of the present study, the disruption of cell–cell interactions are considered to be a key therapeutic mechanism of the anti-VEGFR2 antibody.

Presently, Atezolizumab/Bevacizumab, which is positioned as the first-line drug treatment for HCC, aims to activate antitumor immunity by inhibiting the immune checkpoint molecule PD-L1 and to disrupt tumor vasculature and the TME by inhibiting VEGF-A. Although Shimose et al. reported in a retrospective study that the therapeutic effect of ramucirumab as a post-Atezolizumab/Bevacizumab treatment was significantly greater in terms of progression-free survival than when ramucirumab was administered following other therapies,^4^ the implications of VEGFR2 inhibition on AFP-positive malignant HCC after this combination therapy remains unclear. In this study, our investigation using an established mouse HCC syngeneic model demonstrated the significant antitumor effects of the antimouse VEGFR2 antibody DC101, even after primary treatment involving VEGF-A inhibition. A possible mechanism by which DC101 is effective after anti-VEGF-A antibody therapy is inhibition of VEGF-D. In gliosarcoma and colorectal cancer, the resistance mechanism to the anti-VEGF-A antibody bevacizumab involves the upregulation of VEGF-D production.^17^^,^^18^ VEGF-D has also recently been reported to be upregulated in HCC during progressive disease after Atezolizumab/Bevacizumab.^19^ VEGF-D is an important angiogenic factor that can mediate angiogenesis in a manner comparable to VEGF-A,^20^ suggesting that inhibition of VEGF-D-induced angiogenesis by the anti-VEGFR2 antibody may contribute to its antitumor effect.

The enhancement mechanisms of CSCs through VEGF signaling have been reported. In skin papillomas, CSCs localize to the perivascular niche, interact with ECs, and promote cancer stemness via the VEGF-Nrp1 loop. This mechanism is impaired by VEGFR2 inhibition with DC101.^9^ In HCC, the direct enhancement of CSCs via VEGF through VEGFR2/Nanog has been reported.^21^ Considering these findings, the reduction in CSCs observed in this study due to VEGFR2 inhibition is thought to be attributable not only to the disruption of CSC-EC interactions but also to the inhibition of VEGF signaling in CSCs. Further investigation is needed to clarify the detailed mechanisms.

CSCs are thought to create a microenvironment with stromal cells, including ECs, fibroblasts, and immune cells, that supports their properties and survival. In this study, we investigated the effect of VEGFR2 inhibition on the TME constituted by CSCs. Notably, it was revealed that VEGFR2 inhibition not only suppressed CSCs and ECs but also significantly reduced the population of CD8-positive T cells coexpressing the immune exhaustion markers PD1 and TIGIT. Because VEGF signaling contributes to CD8-positive T cell exhaustion by promoting the expression of checkpoint molecules such as PD1 and TIGIT,^22^^,^^23^ VEGFR2 inhibition may be a promising therapeutic target for enhancing antitumor immune activity. The observation that VEGFR2 inhibition improved T cell exhaustion even after combined immunotherapy, including VEGF-A inhibition as primary therapy in HCC, is particularly noteworthy for future therapeutic applications. In an additional analysis of the tumor immune microenvironment using the Mouse FFPE IO panel, DC101 was found to increase the intratumoral infiltration of Ki67-positive CD4-T cells. This finding also supports the notion that VEGFR2 inhibition enhances antitumor immune activity.

In clinical settings, long-term VEGF inhibition has been associated with glomerular injury, and a high incidence of severe ascites has been reported in patients treated with ramucirumab.^24^ However, in this study, no significant glomerular injury or severe ascites was observed. When resistance to atezolizumab/bevacizumab occurs in clinical practice, alternative treatment options include not only ramucirumab but also TKIs that inhibit VEGF signaling. Among TKIs, lenvatinib has recently been reported to be effective in MASLD-related HCC.^25^ Further investigation is needed to determine which VEGF-targeted agent, including ramucirumab, should be selected based on the etiology or biological characteristics of the tumor.

A limitation of the present study is that the therapeutic efficacy and tolerability of anti-VEGFR2 antibody following long-term administration of combination immunotherapy were not evaluated, highlighting the need for further investigation in future studies.

## Conclusion

VEGFR2-targeted therapy not only inhibited angiogenesis but also suppressed CSCs and activated tumor immunity, and thus it might be effective in AFP-positive advanced HCC after Atezolizumab/Bevacizumab.
